# Femoral Hernia with a Twist

**DOI:** 10.1155/2010/650829

**Published:** 2010-08-11

**Authors:** Sudeendra Doddi, Vishal Sagar, Tarun Singhal, Santosh Balakrishnan, Frank Smedley, Prakash Sinha

**Affiliations:** Princess Royal University Hospital, Farnborough Common, Orpington, Greater London BR6 8ND, UK

## 1. Introduction

In England, there were 4500 cases of femoral hernia for the year 2005-2006, accounting for 3.5% of all herniae [1]. The femoral hernial sac contents may be varied: preperitoneal fat, omentum, small bowel, or colon. In rare instances, the appendix can be found in the hernial sac, a reported incidence of 0.8%. Whether inflamed or not, the finding of vermiform appendix inside a hernia sac is known as Amyand's Hernia, and it classically refers to an inguinal hernia. Claudius Amyand (1681–1740), a French refugee in England, was one of the leading surgeons of his day; and held the accolade of being surgeon to George II of England. In 1735 he was the first to note the presence of a perforated appendix within the inguinal hernia sac in an eleven-year-old boy, and he subsequently performed a successful appendicectomy (Hutchinson, 1993; Franko and Sulkowki, 2002). Acute appendicitis in a femoral hernia is a rare, potentially serious complication. The presence of a vermiform appendix in a femoral hernia sac is termed De Garengeot hernia after Rene Jacques Croissant de Garengeot, an 18th century Parisian surgeon, who is quoted in the literature as the first to describe an appendix in a femoral hernia sac.

It presents as an incarcerated or strangulated hernia, and the surgical management is different for the two conditions. Prompt surgery is needed to avoid complications. Awareness of this condition may help to avoid delay in management. 

We report a case of a patient with an acute appendicitis in a right femoral hernia presenting with a painful groin mass.

## 2. Case Study

An independent 94-year-old Caucasian male presented as an emergency with a two-day history of progressive right groin pain. He did not have fever, nausea, anorexia, or obstructive symptoms. He had previously undergone multiple bilateral inguinal hernia repairs. He had history of noninsulin-dependent diabetes, chronic obstructive pulmonary disease, previous pneumonias, and metatarsalgia. Medications included aminophylline, zopiclone, prednisolone, salbutamol, metformin, and montelukast sodium. 

The general, cardiovascular, and respiratory examinations were normal. Abdominal examination revealed a tender lump in the right groin measuring 2 cm in diameter that was irreducible. However, a cough impulse could not be demonstrated, and the overlying skin was not erythematous. The abdomen was scaphoid, soft, and with no guarding or rigidity. Rectal examination was normal.

A full blood count showed Haemoglobin 11.4 g/dL and white blood cells 7.8 × 10^9^/L. Urea, electrolytes, and liver function were reported as normal. ESR was mildly raised at 51, and the CRP was normal. Erect chest and supine abdominal X-rays were also normal. 

At this time, a presumptive diagnosis of irreducible right recurrent inguinal hernia was made. The patient was admitted, kept nil by mouth, and given intravenous fluids and analgesia in preparation for surgery. 

**Figure 1:**
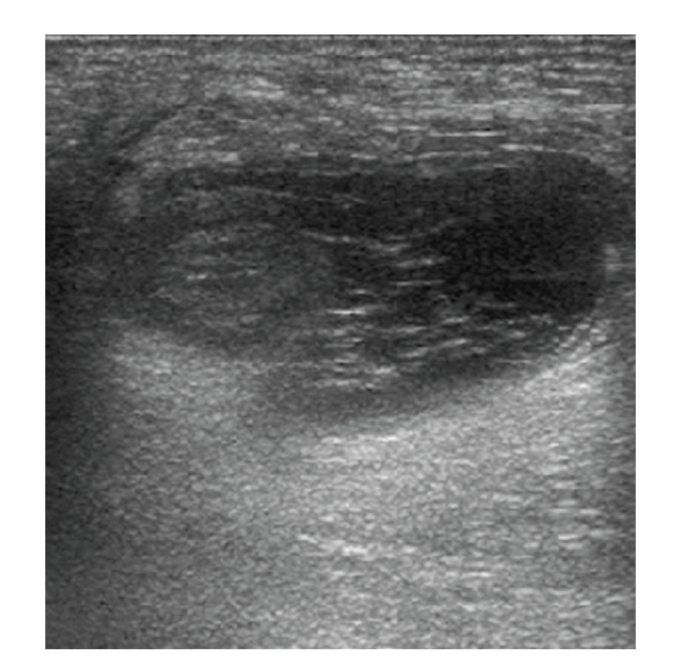
Preoperative ultrasound scan of right groin swelling showing a presumed indirect inguinal hernia containing one bowel loop and fatty omentum.

Ultrasound scan revealed a right indirect inguinal hernia containing one bowel loop and fatty omentum, but it was not possible to determine whether strangulation had taken place (Figure 1).

**Figure 2:**
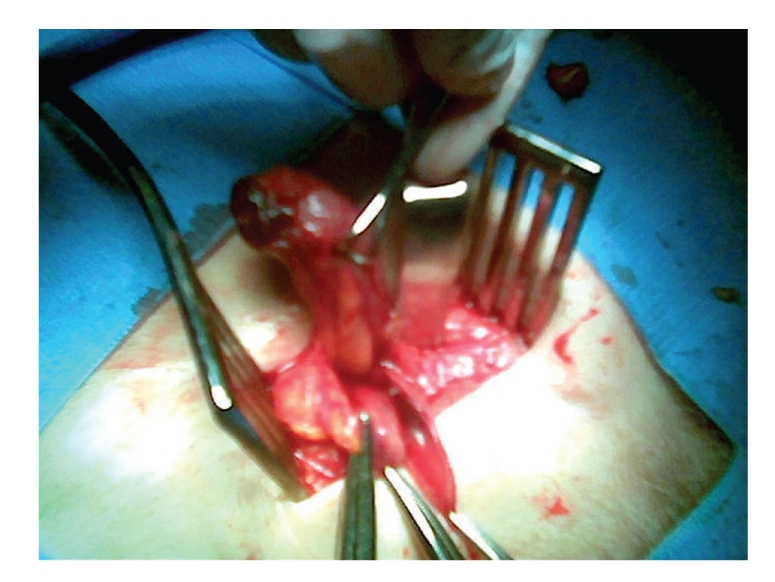
Intraoperative picture from exploration of femoral hernia sac. The acutely inflamed appendix has been delivered out.

The patient underwent surgery under a general anaesthetic. A right groin incision was made; a femoral hernia sac was identified, and dissected by a low approach. On opening the sac, an inflamed, incarcerated appendix was identified (Figure 2). An appendicectomy was performed through the same incision. The femoral canal defect was closed with a primary suture repair. In view of the age of the patient, finding a femoral hernia intraoperatively and an inflamed appendix, a “low approach” was undertaken to repair the femoral hernia. Postoperatively, the patient made an uneventful recovery. He was discharged home three days after surgery. The histology of the appendix confirmed appendicitis.

## 3. Discussion

Femoral herniae account for 4% of all groin herniae and are more common in women and with increasing age [2]. Femoral herniae have the highest rate of incarceration amongst groin herniae, 5%–20% [3], because of the narrow and rigid femoral canal and therefore require early surgical repair. Although most groin masses are simple hernia, occasionally these hernia contain more than just small intestines. Stomach, omentum, colon, and an appendix have all been reported as contents. The latter has an incidence of 0.5%–5% [4]. The appendix is found in the femoral hernial sac more commonly during elective hernia repair than emergency repair [2–5]. The appendix may be in an abnormal anatomical site because of intestinal rotation, variable attachment of caecum, or a large caecum extending into the pelvis [3]. Acute appendicitis within an external hernia is rare—it accounts for 0.13% of all cases of acute appendicitis. Acute appendicitis in a femoral hernia sac is extraordinarily rare—there have been just over 70 cases reported to date [2]. 

Intraabdominal appendicitis is thought to be due to intraluminal obstruction secondary to appendicolith or lymph node hypertrophy. In contrast, appendicitis in a femoral hernia is due to extraluminal compression from a narrow and rigid canal. Probably for this reason appendicitis is more common in femoral herniae than other groin herniae [5]. For the same reason there is no intraperitoneal spread of infection, and patients present with local signs such as erythema and groin tenderness rather than peritonitis.

The commonest presentation is that of a strangulated femoral hernia. It can sometimes present as necrotising fasciitis or small bowel obstruction. The cardinal features of appendicitis are usually absent [6].

Sharma et al. [4], in their retrospective analysis of seven consecutive patients with De Garengeot hernia, found that none of the patients were diagnosed preoperatively, and all patients therefore underwent emergency surgery with a presumptive diagnosis of either incarcerated or strangulated femoral hernia. There are only two case reports of appendicitis in a strangulated femoral hernia being diagnosed pre-operatively on CT scan [3, 7]. Though these two studies considered CT scan to be highly specific and sensitive, routine use of imaging pre-operatively is not feasible because of the rarity of the condition and the need to expedite surgery to avoid complications.

Although well defined and previously discussed, it still baffles most junior surgeons to find an appendix incarcerated within a femoral hernia. There is a need to deal with the content of the hernia, whilst at the same time perform a safe and definitive repair in the face of potential infection due to the nature of the contents. Due to paucity of cases no standard treatment exists. Sharma et al. advocated mesh hernia repair without an appendicectomy in the case of a normal appendix and open appendicectomy followed by sutured hernia repair when the appendix was inflamed. It would be safer to perform an appendicectomy if the appendix is inflamed, perforated, or appearing to be congested. It is feasible in most cases to deliver the appendix out completely through the hernia sac itself after identifying the content and dissecting the strangulated neck of the sac. In cases where mobilisation of the sac and contents is difficult to achieve through a low groin approach, it may be safer to gain additional approach through a low right paramedian incision or cosmetically superior Pfannenstiel incision to achieve reduction and perform an appendicectomy. A normal appendix can however be safely returned into the abdominal cavity followed by a standard mesh or plug-based tension-free hernia repair. 

There is no clear consensus on the use of mesh. Most studies recommend primary tissue repair [4, 5]. However, when there is a groin hernia abscess caused by perforated appendix, some authors advocate incision and drainage with delayed appendectomy and hernia repair [5]. Some authors have mentioned the use of a mesh when the appendix was inflamed in a femoral hernia and reported no wound infection [4, 8]. The main concern is wound infection which is thought to be as high as 29% in view of the delay in diagnosis and old age of the patients [5, 8, 9].

## 4. Conclusion

Incarceration of the vermiform appendix in a femoral hernia is a rare clinical event. Despite the rarity, one should be aware of this condition to minimise morbidity. Appendicectomy is performed after obtaining adequate exposure. The risk of wound infection favours a tissue-based suture repair though in select cases a mesh may be used.
